# MALDI-TOF mass spectrometry as a tool for differentiation of invasive and noninvasive *Streptococcus pyogenes* isolates

**DOI:** 10.1111/j.1574-695X.2008.00428.x

**Published:** 2008-06-05

**Authors:** Hercules Moura, Adrian R Woolfitt, Maria G Carvalho, Antonis Pavlopoulos, Lucia M Teixeira, Glen A Satten, John R Barr

**Affiliations:** 1 Division of Laboratory Sciences, National Center for Environmental Health M.S. F-50, Atlanta, GA, USA; 2 Division of Bacterial Diseases, National Center for Infectious Diseases, Centers for Disease Control and Prevention M.S. F-50, Atlanta, GA, USA; 3 Instituto de Microbiologia, Universidade Federal do Rio de Janeiro Rio de Janeiro, Brazil

**Keywords:** matrix-assisted laser desorption/ionization time of flight mass spectrometry, biomarkers, protein fingerprints, *Streptococcus pyogenes*, ribosomal proteins, necrotizing fasciitis

## Abstract

A novel mass spectral fingerprinting and proteomics approach using MALDI-TOF MS was applied to detect and identify protein biomarkers of group A *Streptococcus* (GAS) strains. *Streptococcus pyogenes* ATCC 700294 genome strain was compared with eight GAS clinical isolates to explore the ability of MALDI-TOF MS to differentiate isolates. Reference strains of other bacterial species were also analyzed and compared with the GAS isolates. MALDI preparations were optimized by varying solvents, matrices, plating techniques, and mass ranges for *S. pyogenes* ATCC 700294. Spectral variability was tested. A subset of common, characteristic, and reproducible biomarkers in the range of 2000–14 000 Da were detected, and they appeared to be independent of the culture media. Statistical analysis confirmed method reproducibility. Random Forest analysis of all selected GAS isolates revealed differences among most of them, and summed spectra were used for hierarchical cluster analysis. Specific biomarkers were found for each strain, and invasive GAS isolates could be differentiated. GAS isolates from cases of necrotizing fasciitis were clustered together and were distinct from isolates associated with noninvasive infections, despite their sharing the same *emm* type. Almost 30% of the biomarkers detected were tentatively identified as ribosomal proteins.

## Introduction

*Streptococcus pyogenes*, frequently referred to as group A *Streptococcus* (GAS), is one of the most common and versatile human bacterial pathogens. It causes a variety of diseases, ranging from mild and quite frequent noninvasive infections of the upper respiratory tract and skin to severe invasive infections that include necrotizing fasciitis and streptococcal toxic shock syndrome (Facklam, 2002). This bacterial species is also associated with such life-threatening poststreptococcal sequelae as acute rheumatic fever and glomerulonephritis. For many years, the evaluation of epidemiologic relationships between GAS isolates was based on serological typing for detection of a bacterial cell surface protein, the M protein, which is considered as a major virulence factor of these microorganisms. More recently, several DNA-based typing methods have been applied to evaluate the diversity of GAS isolates and to elucidate their association with different diseases (Facklam *et al.*, 1999; McGregor *et al.*, 2004; Doktor *et al.*, 2005). A major improvement to serological M typing was *emm* gene sequencing analysis (Beall *et al.*, 1997; Teixeira *et al.*, 2001). However, new typing methods are still needed to improve strain differentiation and to contribute with new insights into the epidemiology and pathogenesis of GAS infections. A better understanding of GAS infections would lead to improvements in the rapid and precise detection of the microorganism and the development of more effective strategies for treatment and prevention of GAS diseases (Currie, 2006).

A practical and rapidly evolving application of mass spectrometry (MS)-based methods in microbiology is the rapid identification of microorganisms and strain differentiation (van Baar, 2000; Fenselau & Demirev, 2001; Lay, 2001). Two soft ionization methods, matrix-assisted laser desorption/ionization-time of flight mass spectrometry (MALDI-TOF MS) and liquid chromatography-electrospray ionization mass spectrometry (LC-ESI MS), are particularly useful in the identification and characterization of proteins in complex mixtures. They play a central role in proteomics research (Aebersold & Mann, 2003; Patterson & Aebersold, 2003; Gingras *et al.*, 2005; Lambert *et al.*, 2005). MS methods offer detection limits superior to traditional methods for the analysis of many relevant microorganisms, in addition to offering other significant advantages. Because of their simplicity, speed, and accuracy, MS methods have been successfully applied for biomarker discovery and for characterizing various bacterial agents (Ruelle *et al.*, 2004; Shaw *et al.*, 2004; Stackebrandt *et al.*, 2005; Valentine *et al.*, 2005; Wunschel *et al.*, 2005a, b; Pignone *et al.*, 2006; Liu *et al.*, 2007). It is now well accepted that the mass spectra obtained by MALDI-TOF MS provide characteristic patterns of proteins (fingerprints composed of unique biomarkers) from whole organisms that can be used to identify bacteria, viruses, protozoa, and fungi (Magnuson *et al.*, 2000; Amiri-Eliasi & Fenselau, 2001; Moura *et al.*, 2003; Krader & Emerson, 2004; Wunschel *et al.*, 2005a). However, interpretation of MS data from complex mixtures is difficult, and early reports using visual analysis of mass spectra confirmed the difficulties in the differentiation of microorganisms (Fenselau & Demirev, 2001; Lay, 2001). Currently, there are two major approaches to identify microorganisms by MS: a fingerprint-statistical-based approach (Holland *et al.*, 1996; Jarman *et al.*, 1999, 2000; Dickinson *et al.*, 2004; Satten *et al.*, 2004; Valentine *et al.*, 2005; Wunschel *et al.*, 2005a, b) and a proteomics approach (Demirev *et al.*, 1999, 2001; Pineda *et al.*, 2000, 2003; Pribil & Fenselau, 2005; Pribil *et al.*, 2005).

Here we report the development and application of MALDI-TOF MS analysis with statistical analysis as a potential complementary method for GAS characterization and strain differentiation. We have applied the MALDI-TOF MS with random forest (rf) analysis, hierarchical cluster analysis, and proteomic database searching to a limited number of invasive GAS and noninvasive GAS isolates. Using a combination of novel data processing methods for visual peak comparison, statistical analysis, and proteomics database searching, we were able to demonstrate the power of this combined approach on a small number of well-characterized and diverse GAS isolates. We believe that the combined approach will strengthen the ability of MALDI-TOF MS to differentiate microorganisms.

## Materials and methods

### Chemicals

All chemicals used during this study were purchased from Sigma-Aldrich (St. Louis, MO) except where indicated. Buffers and culture media were obtained from the Scientific Resources Program at the Centers for Disease Control and Prevention (CDC).

### Bacterial strains

A total of nine GAS strains, comprising the *S. pyogenes* genome strain (ATCC 700294) and eight clinical isolates, were included in this study (Table 1). The panel of selected GAS clinical isolates was obtained from patients with different syndromes who lived in the United States and Brazil. All clinical isolates were collected for previous studies and were obtained using the ethical guidelines of either the CDC or the Instituto de Microbiologia, Universidade Federal do Rio de Janeiro. These isolates were identified previously using conventional phenotypic tests (Facklam, 2002) and were characterized by sequencing of *emm* gene-specific PCR products (*emm*-typing) (Beall *et al.*, 1997; Teixeira *et al.*, 2001). In addition, a reference strain of *Streptococcus agalactiae* (ATCC 13813), another β hemolytic streptococcal species that plays a role as an important agent of infections in humans, was included for comparative purposes. Reference strains of *Enterococcus faecalis* (ATCC 29212), *Escherichia coli* (ATCC 25922), and *Staphylococcus aureus* (ATCC 29213) were included as outgroups for statistical purposes.

**Table 1:** Characteristics of *Streptococcus pyogenes* isolates included in this study

Strain	Source	Origin	*emm* type
Th 892-97	Throat infection	Brazil	*emm1*
Th 834-97	Throat infection	Brazil	*emm1*
Th 1476-97	Throat infection	Brazil	*emm1*
Sk 3961-98	Skin infection	Brazil	*emm12*
Th 3159-00	Throat infection	Brazil	*emm12*
NF 307-03	Necrotizing Fasciitis	USA	*emm1*
NF 312-03	Necrotizing fasciitis	USA	*emm1*
NF 325-03	Necrotizing fasciitis	USA	*emm12*
ATCC 700294	Genome strain	USA	*emm1*

### Growth conditions to obtain bacterial cells for MS analysis

Bacterial strains were initially grown on blood agar (trypticase soy agar containing 5% defibrinated sheep blood) plates at 37 °C for 18–24 h. All microbiological procedures were carried out in a certified Biosafety Level 2 cabinet equipped with high-efficiency particulate air filters. Bacterial cells were inoculated in tubes containing 30 mL of Todd Hewitt broth (THB). After incubation at 37 °C for 18–24 h, cells were washed three times with 10 mL of Tris-sucrose buffer (0.01 M Tris, 0.025 M sucrose, pH 7.0) by centrifugation under refrigeration (4–10 °C). Cells were then suspended in 500 μL of Milli-Q grade water (Millipore) and maintained at −80 °C until they were prepared for MS analysis. To test batch variability, cells from strain ATCC 700294 grown on different occasions over a 3-week (batch 2) and a 6-week (batch 3) interval were prepared and analyzed. The clusters of distinctive peaks or biomarkers desorbed from the first batch of *S. pyogenes* were used as reference spectra to evaluate method reproducibility, batch-to-batch reproducibility, and interference of growth media on biomarker expression and detection. A subset of cells from batch 2 was subjected to 2 × 10^8^ rads of γ irradiation before processing for MALDI-TOF MS analysis. This has been shown to destroy bacterial viability without disrupting the protein structure (Shaw *et al.*, 2004). To test the interference of growth media on biomarker detection, *S. pyogenes* ATCC 700294 was also grown on blood agar plates, and isolated colonies (1, 2, 3, 4, and 5 colonies) were collected and analyzed directly without further treatment. In addition, 30 colonies were pooled, washed three times with Tris-sucrose buffer as described above, and analyzed. The other bacterial samples, including the eight GAS clinical isolates, and the reference strains used throughout the study were cultured three times over a 2-week period and washed as described above.

### Sample preparation

Washed bacterial cells were prepared for MS analysis as described previously (Shaw *et al.*, 2004) with a few modifications. Briefly, whole bacteria were adjusted to concentrations of 10^6^ and 10^7^ organisms μL^−1^ using Milli-Q grade water, and then premixed with equal volumes of the respective matrix solutions just before spotting on the MALDI target. MALDI matrices consisted of 10 mg mL^−1^ saturated solutions of diaminobenzoic acid (DHB), α-cyano-4-hydroxycinnamic acid (CHCA), or 3,5-dimethoxy-4-hydroxycinnamic acid (sinapinic acid; SA). DHB was dissolved in Milli-Q grade water containing 0.1% trifluoroacetic acid (TFA), whereas CHCA and SA were mixed, respectively, with 50% and 70% acetonitrile (ACN) and Milli-Q grade water containing 0.1% TFA. A 192-well stainless-steel sample target plate with numbered circular wells [Applied Biosystems (AB), Framingham, MA] was utilized for MALDI-TOF analysis. The plates were washed with Milli-Q grade water, treated with methanol, and allowed to dry at room temperature. When dry, 0.5 μL of the premixed suspensions containing matrices and whole bacterial forms or mass standards for calibration (Sequazyme Peptide Mass Standards Kit, AB) were spotted in four separate proximal wells to create quadruplicates of samples and controls. Bovine cytochrome *c* (1 mM) was added to one well of each sample as an internal standard. After drying, the plate was inserted into the instrument for MALDI-TOF MS analysis.

### MALDI-TOF MS analysis

Mass spectra were acquired using a MALDI-TOF/TOF mass spectrometer (AB 4700 Proteomics Analyzer) equipped with a nitrogen laser (Nd : YAG) at 337 nm, and with a 200 Hz repetition rate. Analyses were performed on at least three different days in linear-delayed extraction positive ion mode, using an accelerating voltage of 20 kV. For optimum data quality, mass spectra in the 1000–6000 *m/z* range were acquired using either CHCA or DHB as the matrices; for the 2000–14 000 *m/z* range, SA was used as matrix. A minimum of 11 individual spectra representing 10 accumulated subspectra were obtained from each well. The acceptance criteria were based on 1000 laser shots per spot, and they required signal intensities between 2000 and 55 000 counts, with a signal-to-noise of 10 or greater.

### Data processing

Mass spectra from at least four harvestings were processed as follows: profile spectral data were exported as text-format *m/z*-intensity lists with a unified *m/z* scale, using custom Microsoft Visual Basic for Applications (vba) macros in data explorer™, the AB viewing application. The text data were further processed and viewed using a suite of custom Microsoft visual basic.net (vb.net) programs, the vba macros, and vb.net programs ‘multispec processor’ and ‘multispec viewer’. The latter was designed to display hundreds of spectra at once in a number of formats, including a simulated sodium dodecyl sulfate–polyacrylamide gel elelctrophoresis view, making it extremely valuable for visual analysis of the data sets, which may be comprised of several thousand individual spectra. These spectra were normalized to the base peak, smoothed using a 21-point, 2-pass Gaussian algorithm, and finally standardized and denoised using a custom fortran program (Satten *et al.*, 2004). We used past software v1.34 (http://folk.uio.no/ohammer/past/doc1.html) for hierarchical cluster analysis, with the single summed spectra (one spectrum representing each organism) for input. We used RF v5.1 (http://www.stat.berkeley.edu/users/breiman/RandomForests/cc_home.htm) for classification and identification, in this case with nine summed spectra from three separated harvestings (one month apart) of each organism as a training set and three summed spectra as unknowns. The recompilation of the Fortran RF code for each experimental condition was automatically driven by vb.net programs, and custom viewing applications were developed to aid in the interpretation of the RF results.

### Tentative peak matching and database searching

Tentative identification of the prominent peaks was carried out using the Tag−Ident proteomics tool or ExPASy Sequence Retrieval System (http://us.expasy.org). In addition, ‘ms db filter’, a custom vb.net algorithm, was used to construct a CDC-modified database filtered from UNIPROT (http://www.ebi.ac.uk/uniprot/index.html). ms db filter excludes any Swiss-Prot and TrEMBL or UNIPROT entry described as a fragment. It strips out signal and prepeptide sequences and applies a rule to add or remove initial methionine, as described by Pineda (Pineda *et al.*, 2003). The CDC-modified filtered database was used for data mining the deduced proteome from several bacterial species used in this study that have the whole genome sequenced. Custom algorithms within multispec viewer were used to generate peak lists from the acquired mass spectra and to match these peaks with the CDC-modified database to provide tentative protein identifications. The extracted peak lists were also submitted to the ‘Rapid Microorganism Identification Database’ (http://pinedalab.jhsph.edu/microOrgID).

## Results

### *Streptococcus pyogenes* ATCC 700294 baseline spectra and effect of bacterial growth media on biomarker detection

**Fig. 1:**
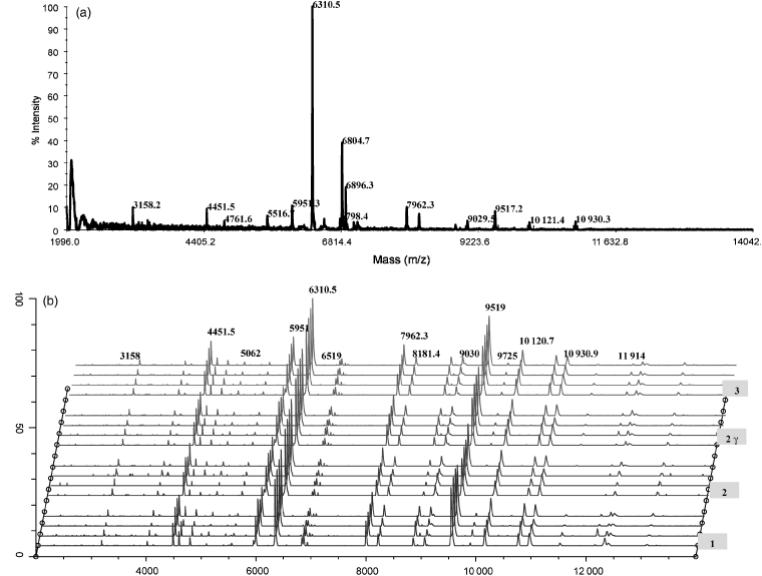
MALDI-TOF mass spectra (*m/z* 2000–14 000 Da) for *Streptococcus pyogenes* ATCC 700294 whole cells grown in liquid media (THB). (a) Represents typical raw mass spectra of 10^7^ cells. (b) Depicts the reproducible patterns in denoised spectra revealed using multispec viewer after analyzing several runs of *S. pyogenes* (batches 1, 2, and 3), including a subset from batch 2 that was γ irradiated. Analyses were in quadruplicate in each case.

**Fig. 2:**
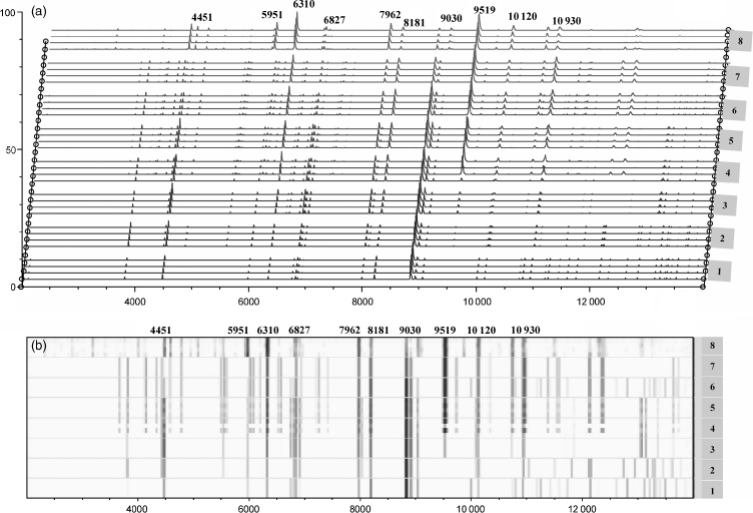
multispec viewer depicting the influence of different colony numbers and culture media (blood agar and THB) in spectra variability of *Streptococcus pyogenes* ATCC 700294. (a) Mass spectra. Annotated denoised peaks are consistently seen in all wells. (b) Gel view feature to the mass spectra. From bottom to top, numbers 1, 2, 3, 4, 5, and 6 denote groups of four spectra obtained, respectively, from 1, 2, 3, 4, 5, and 30 colonies of *S. pyogenes* grown on blood agar and directly plated onto the MALDI target; numbers 7 and 8 are spectra obtained from washed cells from 30 colonies grown on blood agar (7) and 10^8^ cells grown in THB (8).

Three batches of *S. pyogenes* genome strain grown in liquid media were analyzed by MALDI-TOF MS. Spectra obtained on 10^6^–10^8^ whole bacteria μL^−1^ revealed complex spectral patterns with 20–40 peaks in the *m/z* range corresponding to 1000–6000 Da or 2000–14 000 Da. Observed mass spectra are believed to consist primarily of protonated peptide and protein signals, although some signals below *m/z* 4000 may represent other materials. The mass range corresponding to 2000−14 000 Da was preferred for this study because the peaks in this region were more abundant and more consistent. A representative example of *S. pyogenes* mass spectra is shown in Fig. 1a, while Fig. 1b depicts the reproducible patterns from denoised summed spectra revealed using multispec viewer. A γ-irradiated subset from batch 2 is included in Fig. 1b and shows few changes in the summed spectra as compared with the MALDI-TOF spectra obtained on whole live organisms. Visual analysis of processed spectra using multispec viewer revealed only a minor variation from day to day and from well to well in the quadruplicate analysis of the suspensions containing 10^6^ or 10^7^ bacterial cells. Figure 2 shows the spectra and a gel view representation of the mass spectra obtained from a single colony and from pools of 2, 3, 4, 5, and 30 colonies of *S. pyogenes* grown on blood agar and loaded directly onto the MALDI target. These spectra showed altered peaks, along with a set of similar but noisier peaks when compared with those obtained from cells grown in liquid media. Although a higher level of background noise was present in cells grown on blood agar, a key set of peaks corresponding to *m/z* 4451, 5951, 6310, 7962, 8181, and 9030 and representing potential biomarkers were consistently seen in all wells, as is easily observed in the denoised spectra depicted in Fig. 2.

### multispec viewer revealed differences among genus and species

**Fig. 3:**
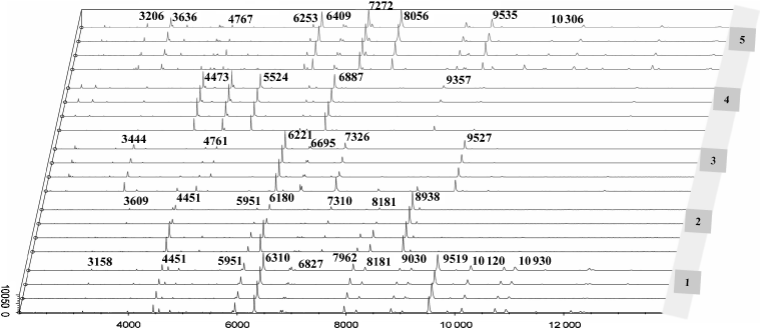
multispec viewer of MALDI-TOF denoised mass spectra (*m/z* 2000–14 000 Da) for whole cells of (1) *Streptococcus pyogenes* ATCC 700294, (2) *Streptococcus agalactiae*, (3) *Enterococcus faecalis*, (4) *Staphylococcus aureus*, and (5) *Escherichia coli*, revealing differences among genus and species. Quadruplicate analyses are shown for each organism.

Three batches of washed cells of *S. agalactiae* (ATCC 13813), *E. faecalis* (ATCC 29212), *S. aureus* (ATCC 29213), and *E. coli* (ATCC 25922) revealed typical spectral patterns in the *m/z* range of 2000−14 000 Da. Figure 3 depicts spectra of the three bacterial standards as compared with *S. pyogenes* ATCC 700294, and *S. agalactiae* ATCC 13813. Bacterial isolates belonging to the same genera and species and grown under the same conditions displayed similar, reproducible sets of peaks such as peaks at *m/z* 4451, 5951, and 8181 for *S. pyogenes* and *S. agalactiae*. In addition, a different pattern was revealed for each species, and these differences include peaks at *m/z* 6221, 7326, and 9527 for *E. faecalis*, peaks at *m/z* 4473, 5524, and 6887 for *S. aureus*, and peaks at *m/z* 6409, 7272, and 8056 for *E. coli*.

### Statistical analyses improved interspecies and strain discrimination in *Streptococcus*

**Fig. 4:**
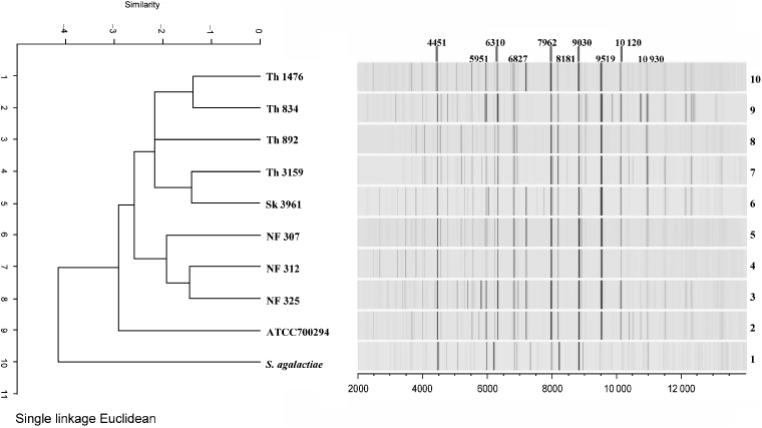
Dendrogram obtained with past (http://folk.uio.no/ohammer/past/index.html) and corresponding gel view of mass spectra using multispec viewer, where differences between *Streptococcus agalactiae* and *Streptococcus pyogenes* could be detected visually. GAS isolates were clustered together and subclusters of invasive and noninvasive isolates of *S. pyogenes* could be detected. For this analysis, all spectra for each strain were summed to give one representative spectrum per organism.

Differences between two species within the genus *Streptococcus* can be easily seen in Fig. 3. The peaks corresponding to *m/z* 3158, 6310, and 7962 were unique for *S. pyogenes*, while peaks corresponding to *m/z* 3609, 6180, and 7310 were unique for *S. agalactiae*. However, we preferred to use a statistical analysis of the MALDI-TOF MS data to quickly reveal significant differences among the strains analyzed. Streptococcal strains could be discriminated from each other by rf (Supplementary Fig. S1). Additionally, cluster analysis with past showed similarities in spectra and was used to cluster species and strains on the basis of these spectral similarities (Fig. 4). The RF analysis (Supplementary Fig. S1) yielded an estimated overall classification error of 1.7%, and the RF algorithm successfully classified all the bacterial species and the majority of the GAS strains in the training set. However, the RF algorithm was unable to differentiate a subset of *emm*1 GAS strains isolated from patients with throat infection, and the estimated classification error for this subset was 22%. Results with three clustering algorithms within past, such as paired group, single linkage, and Ward's method, using similarity measure methods such as Euclidean, Dice, and Jaccard, among others, were analogous and quite consistent regardless of the analysis method applied, so that the different bacterial species and strains studied could be reliably and reproducibly separated from each other (Fig. 4). As expected, GAS isolates were clustered together, and even subclusters could be detected, including the three strains isolated from necrotizing fasciitis cases that were grouped together, and were separate from noninvasive GAS isolates.

### Tentative identification of peaks by database search

The peak lists generated using multispec viewer were compared with the CDC-modified filtered protein database; 97 tentative matches were uncovered, including several peaks listed as ribosomal proteins. A total of 21 ribosomal proteins detected in the MALDI-TOF mass spectra of each GAS strain analyzed are listed in Table 2. Tentative matches from the CDC-modified database filtered to select ribosomal proteins were overlaid with a typical summed spectrum of *S. pyogenes* (Supplementary Fig. S2). In addition, subsets of summed *m/z* values of biomarkers detected from the first batch of *S. pyogenes* listed in Table 2 served as reference standards to evaluate both their batch-to-batch reproducibility and interference of growth media on biomarker expression. Similar and consistent results were obtained with processed peak lists of *E. coli* strain ATCC 25922, *S. aureus* strain ATCC 29213, and *E. faecalis* strain ATCC 29212, where many of the tentatively identified peaks also corresponded to ribosomal proteins (data not shown). The peak lists submitted to the ‘Rapid Microorganism Identification Database’ returned correct matching results for each genus/species tested.

**Table 2:**
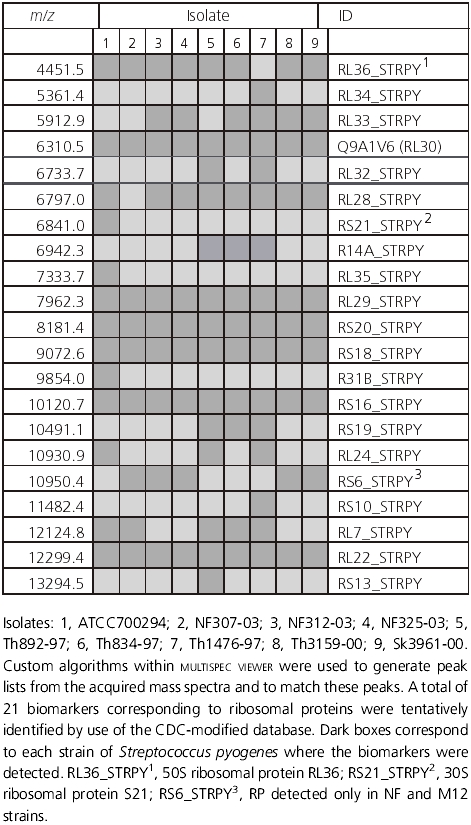
Ribosomal proteins detected in the MALDI-TOF MS of each *Streptococcus pyogenes* strain analyzed

## Discussion

MALDI-TOF MS detection of biomarkers desorbed from whole cells is an emerging technique potentially applicable to the identification of microorganisms at different levels (van Baar, 2000; Fenselau & Demirev, 2001; Lay, 2001). An increasing number of reports have shown the successful use of fingerprinting by comparing statistically processed mass spectra with a reference database (Jarman *et al.*, 1999, 2000; Dickinson *et al.*, 2004; Satten *et al.*, 2004; Valentine *et al.*, 2005; Wunschel *et al.*, 2005a, b). Other reports indicate that robust identification can be achieved on the basis of proteome database queries (Demirev *et al.*, 1999, 2001; Pineda *et al.*, 2000, 2003; Pribil & Fenselau, 2005; Pribil *et al.*, 2005). In a recent report, consistently correct identifications were obtained when only the most abundantly expressed proteins (e.g. ribosomal proteins) were used in the database (Pineda *et al.*, 2003). Once the MALDI-TOF MS spectra are collected, multiple forms of data analysis can be carried out on these data to discriminate the genus, species, and even strains of the microorganisms.

In the present study, using a high-resolution MALDI-TOF MS instrument along with a combination of mass spectral visual analysis, fingerprinting, and the proteomics approach, we were able to detect and tentatively identify protein biomarkers of nine selected GAS isolates, including *S. pyogenes* strain ATCC 700294, the genome strain. Processed spectra were used for visual and statistical analyses, and they were used to query proteome databases, including a CDC-modified filtered database.

Reproducibility of MALDI-TOF MS spectra is an important concern that has been addressed in a number of studies (Gantt *et al.*, 1999; Saenz *et al.*, 1999; Evason *et al.*, 2000; Jarman *et al.*, 2000; Fenselau & Demirev, 2001; Valentine *et al.*, 2005; Wunschel *et al.*, 2005a, b). In the present study, different MALDI preparations were initially tested using ATCC 700294 and varying solvents, matrices, plating techniques, and mass ranges. Spectral variability was analyzed using the bacterial cells grown under different conditions and culture media. Overall, the spectra of replicate samples were similar, even spectra acquired from wells with different concentrations of bacteria or harvested on different days and from various culture media. γ Irradiation did not seem to alter peaks detected by MALDI-TOF MS and is an alternative to process virulent isolates before analysis by mass spectrometry. A common subset of biomarkers was found consistently with all the conditions studied, and these biomarkers were consistent with previously reported studies (Valentine *et al.*, 2005). MALDI-TOF MS patterns of *S. pyogenes* were reproducible within sets of replicates and over time, thus indicating the potential of this method as a sensitive, specific, and rugged assay for *S. pyogenes* analysis.

The usefulness of the combined algorithms for data processing applied in this study, along with previously reported methods for spectral standardizing and denoising (Satten *et al.*, 2004), was demonstrated by the robust differentiation by rf of all species and most of the strains tested. In addition, plotted peaks pointed to the existence of strain-specific biomarkers. This plot can be used to determine which peaks should be investigated further for biomarker identification by sequencing. Dendrograms obtained with the past cluster software confirmed the results from rf, indicating the potential of MALDI-TOF MS for discrimination among genus, species, and strains.

The genome of at least 12 strains of *S. pyogenes* has been completely sequenced; thus, tentative peak identification was easily accomplished using the CDC-modified filtered protein entries from Swiss-Prot/TrEMBL and UNIPROT databases. Our finding that the several peaks tentatively identified corresponded to ribosomal proteins is intriguing because it points to another advantage of the method as it uncovers several important gene products in only one experiment. Ribosomal proteins are extremely ancient molecules, and one-third of ribosomal protein families are conserved among *Bacteria*, *Archae*, and *Eucharia* (Lecompte *et al.*, 2002). In addition, our findings pointed to the usefulness of small protein analysis in the range of 2000–14 000 Da. These small proteins have not been explored in proteomic studies using two-dimensional gel electrophoresis to detect differences/similarities among organisms (Patterson & Aebersold, 2003; Lambert *et al.*, 2005), but they are promising biomarkers, as the sequenced genomes of the *S. pyogenes* isolates (http://www.ncbi.nlm.nih.gov/entrez/) predict an average of 650 proteins in the 2000–14 000 Da molecular mass range, a third of them with an estimated pI 7.0 or higher.

MALDI-TOF MS-based methods are very sensitive and have been used before for genus and species differentiation, but they have rarely been used for strain characterization (Stackebrandt *et al.*, 2005). Here we report one of the first uses of MALDI-TOF MS for analyzing GAS strains that were discriminated by this method although they were closely related by a genetic typing (*emm* typing), traditionally used for tracing GAS isolates. Additionally, once MALDI-TOF MS spectra are collected, they can be reanalyzed at a later date as more species and strains have their genomes sequenced or after more advanced statistical analyses become available.

GAS strains have been analyzed by different methods, but each has limitations, and thus far, all have failed to differentiate strains according to their sources (invasive and noninvasive) (Beres *et al.*, 2006). In the present work, invasive GAS isolates from cases of necrotizing fasciitis were clustered together, yet separate from isolates associated with mild, noninvasive throat and skin infections, despite sharing the same *emm* type. There was no correlation between the *emm* type and the data obtained by mass spectrometry. We plan to further investigate the consistency of these findings using a larger number of strains isolated from patients with necrotizing fasciitis and will investigate the possibility of disclosing the existence of a subset of biomarkers common to these invasive isolates, whose identity can be further confirmed by sequencing with tandem mass spectrometry analysis (MS/MS). These initial results suggest that specific biomarkers can be used for early detection of the disease. GAS is the most common causative agent of necrotizing fasciitis, a rare but disfiguring and frequently lethal condition. Analysis of the nine selected GAS strains suggests the existence of a group of unique spectral markers that can be useful to characterize GAS isolated from NF cases. To our knowledge, this is the first report with consistent evidence of biomarker differences among invasive and noninvasive GAS isolates.

In conclusion, we have developed a methodology for proteomic analysis of bacteria using MALDI-TOF MS with statistical analysis. Visual analysis of multiple spectra was sufficient to differentiate among the different genera and species analyzed. However, the combined MS visual analysis, fingerprinting, and proteomics approach yielded a more robust biomarker identification using a database search, which allowed strain differentiation among GAS isolates, including the separation of a group of isolates that caused necrotizing fasciitis from another group of isolates associated with noninvasive infections. Small ribosomal proteins were consistently found among biomarkers detected in this study. Even though the current study used a limited number of carefully selected, well-characterized, and diverse group of GAS isolates, we believe that the data suggest that MALDI-TOF MS may constitute a powerful tool to distinguish GAS isolates and to investigate virulence-related characteristics, reinforcing the concept that this method is a rapid, reproducible, high-throughput alternative for the characterization of microorganisms.

## Statement

Reuse of this article is permitted in accordance with the Creative Commons Deed, Attribution 2.5, which does not permit commercial exploitation.

## Supplementary material

The following Supplementary material is available for this article online:

**Fig. S1.** Results of classification of datasets using RF.

**Fig. S2.** Tentative protein identification using the CDC-modified database.

This material is available as part of the online article from: http://www.blackwell-synergy.com/doi/abs/10.1111/j.1574-695X.2008.00428.x (This link will take you to the article abstract).
