# Supplementary material for: MALDI-TOF mass spectrometry as a tool for differentiation of invasive and noninvasive Streptococcus pyogenes isolates
Source: FEMS Immunol Med Microbiol. 2008 Jun 5;53(3):333–42. doi: 10.1111/j.1574-695X.2008.00428.x (PMC2658026; doi:10.1111/j.1574-695X.2008.00428.x)
Supplement: Supplementary file 1 [file fim0053-0333-SD1.pdf]

Fig. S1

Standards	TrueCls	ATCC700204	NF 307- 03	NF 312- 03	NF 325- 03	Th 829- 97	Th 834- 97	Th 1476- 97	Th 3159- 00	Sk 3961- 98	Sagalac	Entero	Staph	Ecoli	TrueCls
InputCls	Cl-#	1	2	3	4	5	6	7	8	9	10	11	12	13	Totals
ATCC700204	1	9													9
NF 307- 03	2		9												9
NF 312- 03	3			9											9
NF 325- 03	4				9										9
Th 829- 97	5					8	1								9
Th 834- 97	6						7								7
Th 1476- 97	7						1	9							10
Th 3159- 00	8								9						9
Sk 3961- 98	9									9					9
Sagalac	10										9				9
Entero	11											9			9
Staph	12												9		9
Ecoli	13													9	9
InputCls	Totals	9	9	9	9	8	9	9	9	9	9	9	9	9	116
Errors(%)	1.7	0%	0%	0%	0%	0%	22.2	0%	0%	0%	0%	0%	0%	0%	Stds

Fig. S2

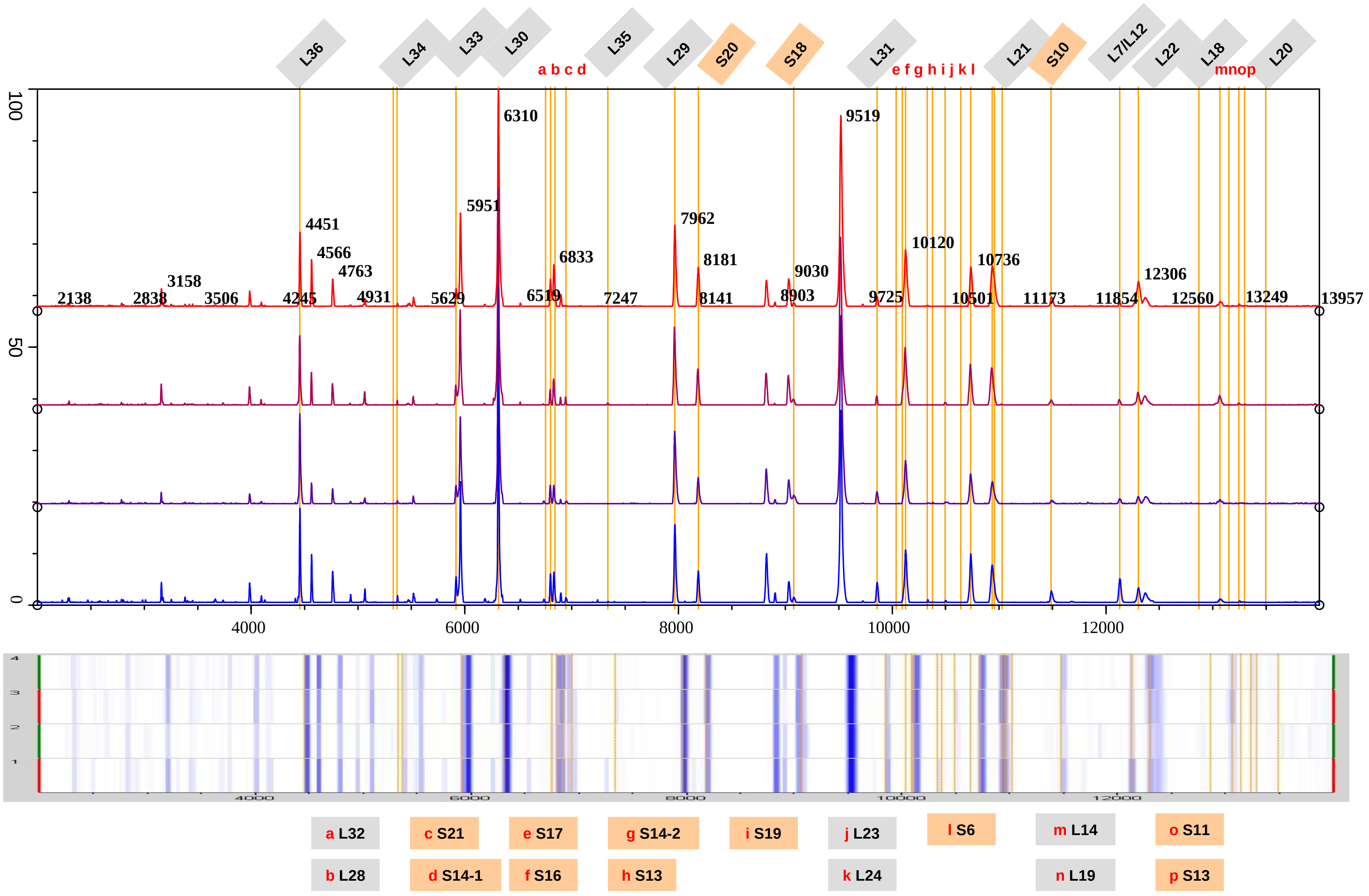

L36
L34
L33
L30
L35
L29
S20
S18
L31
L21
S10
L7/L12
L22
L18
L20
a b c d
e f g h i j k l
mnop
100
50
0
6310
9519
5951
4451
7962
4566
10120
6833
8181
10736
4763
9030
12306
3158
2138
2838
3506
4245
4931
5629
6519
7247
8141
8903
9725
10501
11173
11854
12560
13249
13957
4000
6000
8000
10000
12000
a L32
b L28
c S21
d S14-1
e S17
f S16
g S14-2
h S13
i S19
j L23
k L24
l S6
m L14
n L19
o S11
p S13
